# Long-Term Administration of Conjugated Estrogen and Bazedoxifene Decreased Murine Fecal β-Glucuronidase Activity Without Impacting Overall Microbiome Community

**DOI:** 10.1038/s41598-018-26506-1

**Published:** 2018-05-25

**Authors:** Karen Lee Ann Chen, Xiaoji Liu, Yiru Chen Zhao, Kadriye Hieronymi, Gianluigi Rossi, Loretta Sue Auvil, Michael Welge, Colleen Bushell, Rebecca Lee Smith, Kathryn E. Carlson, Sung Hoon Kim, John A. Katzenellenbogen, Michael Joseph Miller, Zeynep Madak-Erdogan

**Affiliations:** 1 Division of Nutritional Sciences, University of Illinois, Urbana-Champaign, Urbana, IL USA; 2 Department of Food Science and Human Nutrition, University of Illinois, Urbana-Champaign, Urbana, IL USA; 3 Department of Pathobiology, University of Illinois, Urbana-Champaign, Urbana, IL USA; 4 National Center for Supercomputing Applications, University of Illinois, Urbana-Champaign, Urbana, IL USA; 5 Carl R. Woese Institute for Genomic Biology, University of Illinois, Urbana-Champaign, Urbana, IL USA; 6 Carle Illinois College of Medicine, University of Illinois, Urbana-Champaign, Urbana, IL USA; 7 Department of Chemistry, University of Illinois, Urbana-Champaign, Urbana, IL USA; 8 Cancer Center at Illinois, University of Illinois, Urbana-Champaign, Urbana, IL USA; 9 Beckman Institute for Advanced Science and Technology, University of Illinois at Urbana-Champaign, Urbana, IL USA; 10 Present Address: The Roslin Institute, University of Edinburgh, Edinburgh, Scotland UK

## Abstract

Conjugated estrogens (CE) and Bazedoxifene (BZA) combination is used to alleviate menopause-associated symptoms in women. CE+BZA undergo first-pass-metabolism in the liver and deconjugation by gut microbiome via β-glucuronidase (GUS) enzyme inside the distal gut. To date, the impact of long-term exposure to CE+BZA on the gut microbiome or GUS activity has not been examined. Our study using an ovariectomized mouse model showed that CE+BZA administration did not affect the overall cecal or fecal microbiome community except that it decreased the abundance of *Akkermansia*, which was identified as a fecal biomarker correlated with weight gain. The fecal GUS activity was reduced significantly and was positively correlated with the abundance of Lactobacillaceae in the fecal microbiome. We further confirmed in *Escherichia coli* K12 and *Lactobacillus gasseri* ADH that Tamoxifen-, 4-hydroxy-Tamoxifen- and Estradiol-Glucuronides competed for GUS activity. Our study for the first time demonstrated that long-term estrogen supplementation directly modulated gut microbial GUS activity. Our findings implicate that long-term estrogen supplementation impacts composition of gut microbiota and microbial activity, which affects estrogen metabolism in the gut. Thus, it is possible to manipulate such activity to improve the efficacy and safety of long-term administered estrogens for postmenopausal women or breast cancer patients.

## Introduction

Estrogen supplementation/Estrogen Replacement Therapy (ERT) has been used to improve menopause-associated symptoms, including hot flashes and osteoporosis. Data from postmenopausal women and animal studies show that ERT also improves weight gain, dyslipidemia, hypertension and insulin resistance caused by low estrogens^1–3^. However, excess estrogen in the circulation, mainly produced from adipose tissue during menopause, can contribute to hormone-induced malignancies^4,5^. For example, the high ratio of circulating estrogen metabolites/parent estrogens has been linked to increased risk for postmenopausal estrogen receptor positive (ER+) breast cancer^6^.

One current ERT, Conjugated Estrogens and Bazedoxifene (CE+BZA), binds to estrogen receptors (ER) and has tissue selective effects. CE are agonists of ERα while BZA is an agonist in certain estrogen-sensitive tissues such as bone, but acts as an antagonist in others, such as the uterus^7^. CE+BZA is currently used to alleviate postmenopausal symptoms without increasing cancer risk. In cell culture experiments, CE were much less potent than estradiol (E2) in inducing breast cancer cell proliferation, and BZA entirely suppressed such effect^8^. The combination also decreased both uterine and mesenteric white adipose tissue weight without increasing overall uterine weight^9^. In addition, the CE+BZA combination decreased serum leptin levels, leptin/adiponectin ratio, and thiobarbituric acid reactive substances in female mice, preventing fat accumulation and improved insulin resistance and glucose intolerance without stimulating uterine growth^10^. Our previous study using a well-established estrogen deficiency model^11–14^ showed that CE+BZA treatment decreased weight gain after ovariectomy and improved liver steatosis by regulating gene expression programs associated with liver lipid metabolism^15^. In addition, we reported that serum glucuronic acid – a product of deconjugation of estrogens- levels were decreased in mice, leading to our hypothesis that CE+BZA may impact the metabolism of estrogens in the gut.

Estrogens undergo several stages of metabolism inside the body. The CE are well absorbed from the gastrointestinal tract, while BZA is poorly absorbed^16^. After first-pass metabolism in liver, the endogenous and exogenous estrogens go through enterohepatic circulation from the liver, to the bile^17^, and finally into the small intestine. In the liver, estrogens are conjugated through glucuronidation or sulfation before being excreted back into the intestine^18^. In the intestine, conjugated estrogens are deconjugated by the gut microbiome and are partially reabsorbed^19,20^. The deconjugation process, owing at least partially to the microbial β-glucuronidase (GUS) enzymatic activity, influences the half-life and efficacy of ERT drugs^21^.

GUS and similar enzymes have been found in many gut bacterial families, including Bacteroidaceae, Bifidobacteriaceae, Clostridiaceae, Enterobacteriaceae, Lactobacillaceae, Ruminococcaceae and Verrucomicrobiaceae^22^. Studies have correlated bacterial diversity of fecal microbiome to systemic estrogen levels^21^ and estrogen metabolites^23^ in pre- and postmenopausal women. One study reported that both a diet rich in estrogenic isoflavones and the host estrogen receptor status affected the composition of gut microbiome in mice^24^. However, whether prolonged exposure to synthetic estrogens, which is often the case for ERT use, would impact the gut microbiome composition and activity, has not been elucidated.

To understand the impact of long-term estrogen administration on the gut microbiome, we used an established estrogen-deficiency mouse model to examine the effect of E2, CE and BZA alone or in combination. We examined the cecal and fecal microbiome following each treatment and measured GUS activity in fecal microbiota. We performed substrate competition studies for the GUS activity in *Escherichia coli* K12 and *Lactobacillus gasseri* ADH using several estrogen conjugates. In addition, we used machine learning with Extended Random Forest (ERF) algorithm^25–27^ to integrate microbiome and metabolome data from the same study^15^ to identify fecal bacterial biomarkers for the effectiveness of ERT.

## Results

### CE+BZA combination did not affect overall microbiota diversity but decreased GUS activity in fecal microbiome

**Figure 1:**
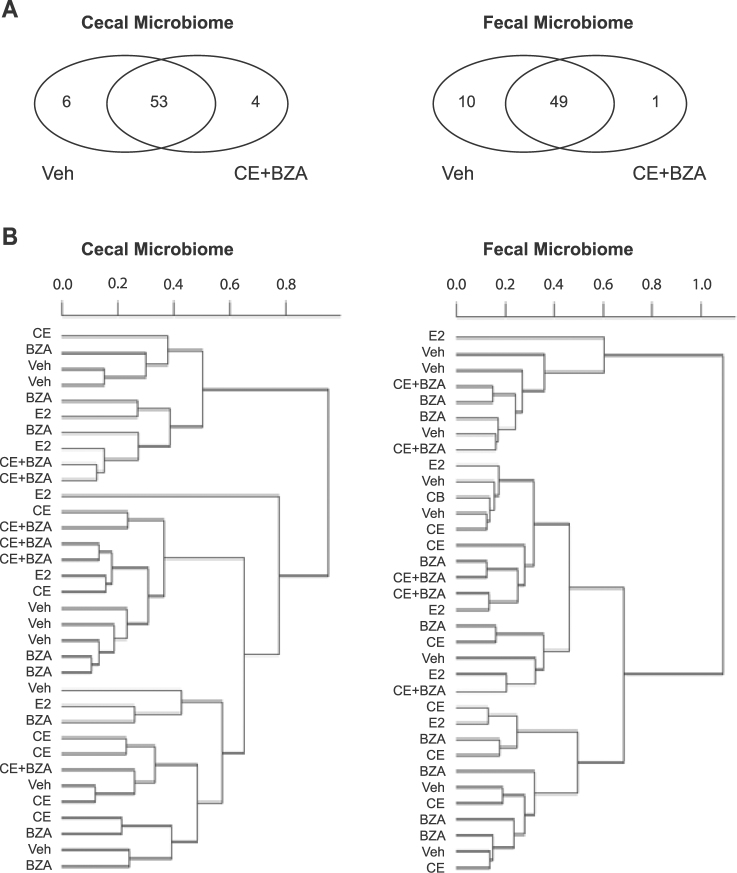
(**A**) Venn diagram comparing the observed bacterial genera in the cecal and fecal microbiome of mice after Veh or CE+BZA treatment. (**B**) Clustering analysis based on weighted Unifrac distances of cecal and fecal microbiome of mice treated with: Veh - vehicle (n = 8); E2 - estradiol (n = 5); CE - conjugated estrogen (n = 7); BZA - Bazedoxifene (n = 8); CE+BZA - conjugated estrogen combined with Bazedoxifene (n = 6).

**Figure 2:**
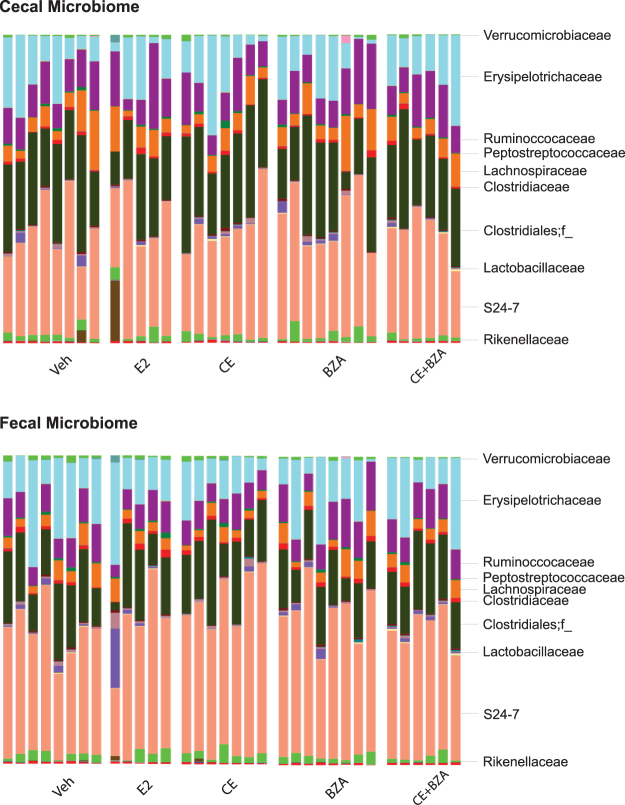
Top 10 most abundant bacterial families (percentage total) in the cecal and fecal microbiome of each mouse treated with: Veh - vehicle (n = 8); E2 - estradiol (n = 5); CE - conjugated estrogen (n = 7); BZA - Bazedoxifene (n = 8); CE+BZA - conjugated estrogen combined with Bazedoxifene (n = 6).

To understand how estrogen administration modulated the community structure of the gut microbiome, we performed microbiome analysis using cecal and fecal samples from ovariectomized female mice that were supplemented with Vehicle (Veh as negative control) and various estrogens including E2 (5 µg.kg^−1^.day^−1^), Conjugated estrogens (CE, 2.5 mg.kg^−1^.day^−1^), Bazedoxifen (BZA, 3 mg.kg^−1^.day^−1^) and CE+BZA for six weeks. Overall, the bacterial genera shared between Veh and CE+BZA accounted for >99% of the total bacterial abundance (Fig. 1A). The 10 genera unique to Veh or CE+BZA treatment only accounted for a very small fraction of the total cecal/fecal microbiome. In the cecal microbiome, the six genera unique to Veh added up to less than 0.02% of the total abundance and the four genera unique to CE+BZA added up to less than 0.01% abundance of the total bacterial abundance. In the fecal microbiome, the 10 genera unique to Veh added up to less than 0.02% abundance and the one genus unique to CE+BZA added up to less than 0.03% abundance of the total bacterial abundance. Further analysis based on weighted UniFrac distances showed no clustering following different treatments in either cecal or fecal microbiome (Fig. 1B). One-way ANOVA analysis showed no significant change in the abundance of bacterial families following different estrogen treatments (Fig. 2). At the genus-level, the abundance of *Akkermansia* decreased following CE+BZA compared to Veh (P = 0.0277). We performed Pearson correlation analysis on the abundance of bacterial genera between the cecal and fecal microbiome data. The bacterial genera with a significant correlation (P = 0.002) are listed in Table S1. These bacterial genera were from a variety of phyla including *Actinobacteria*, *Bacteroidetes*, *Firmicutes*, *Proteobacteria* and *Verrucomicrobia*.

Alpha-diversity analyses of the cecal and fecal microbiome included the number of observed Operational taxonomical units (OTU), Shannon index, and Simpson index. No statistically significant change was found in these indices between the different treatment groups (Table 1).

**Table 1:** Alpha-diversity of the cecal and fecal microbiome of mice.

Treatment^a^	Cecal Microbiome^b^				Fecal Microbiome^b^			
	Chao1	Observed OTUs	Shannon	Simpson	Chao1	Observed OTUs	Shannon	Simpson
Veh	1032.16 ± 157.86	661.00 ± 82.03	5.84 ± 0.67	0.92 ± 0.04	933.40 ± 71.93	599.13 ± 36.64	5.47 ± 0.28	0.91 ± 0.02
E2	1004.41 ± 141.80	631.00 ± 103.98	5.82 ± 0.64	0.93 ± 0.02	830.59 ± 155.76	549.60 ± 82.20	5.17 ± 0.50	0.90 ± 0.05
CE	1012.14 ± 113.78	626.00 ± 87.25	5.56 ± 0.71	0.91 ± 0.06	808.13 ± 86.49	529.71 ± 54.80	5.04 ± 0.40	0.89 ± 0.04
BZA	1008.92 ± 124.60	638.50 ± 60.61	5.97 ± 0.40	0.94 ± 0.02	928.27 ± 141.84	594.00 ± 91.27	5.50 ± 0.56	0.92 ± 0.02
CE+BZA	1048.23 ± 85.78	655.83 ± 72.51	5.78 ± 0.35	0.93 ± 0.01	979.08 ± 71.97	629.33 ± 32.20	5.63 ± 0.28	0.92 ± 0.02

^a^Microbiota from mice treated with: Veh - vehicle (n = 8); E2 - estradiol (n = 5); CE - conjugated estrogen (n = 7); BZA - Bazedoxifene (n = 8); CE+BZA - conjugated estrogen combined with Bazedoxifene (n = 6).

^b^Microbial analysis by Illumina 16S rRNA sequencing (V3-V5 hypervariable region). Rarefaction was calculated based on 9910 seqs per sample.

**Figure 3:**
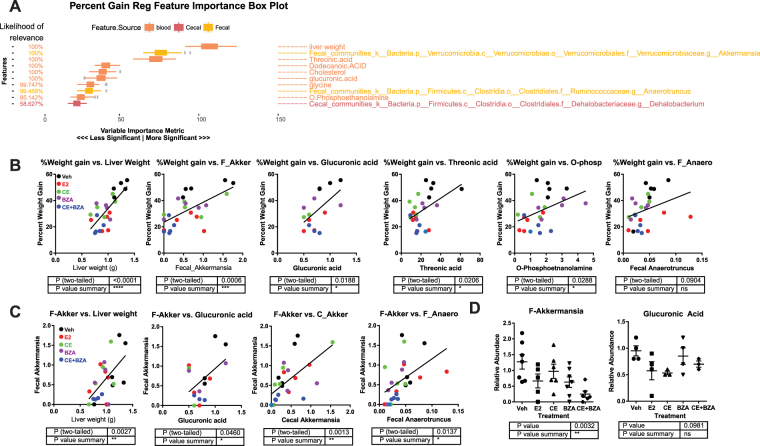
(**A**) Identification of important features including fecal bacterial taxa and plasma metabolites that correlate with lowest% weight gain. Detailed association data for samples (n = 4) were shown for several features’ association against (**B**) weight gain and *Akkermansia*. (**C**) Correlation between the abundance of fecal *Akkermansia*, *Anaerotruncus* and blood glucuronic acid levels, liver weight and percent weight change. Two-tailed pairwise t-tests were performed to identify correlation between indicated factors, metabolites or bacteria level. *P < 0.05, **P < 0.01, ***P < 0.001. (**D**) The levels of *Akkermansia* and glucuronic acid following each treatment. Statistical significance was established at α = 0.05. One-way Anova with a Dunn’s correction were used to identify treatments that were significantly different from Veh. *P < 0.05, **P < 0.01, ***P < 0.001.

Since our previous study showed CE+BZA treatment decreased serum glucuronic acid in the mice^15^, we decided to use machine learning with ERF algorithm to integrate microbiome and metabolite datasets^15^ to obtain a deeper understanding how the gut microbiome impacts the metabolism of different estrogens. The ERF analysis identified liver weight, two fecal bacterial genera-*Akkermansia* and *Anaerotruncus* and several metabolites including glucuronic acid as features that could discriminate between individuals with low and high percent weight gain after ovariectomy and estrogen supplementation (Fig. 3A). The abundance of *Akkermansia* in the fecal microbiome was positively correlated with percent weight change **(**Fig. 3B**)** and liver weight **(**Fig. 3C**)** and the samples were clustered based on the treatment groups. The abundance of *Akkermansia* was decreased in the CE+BZA group compared to the Veh group (p = 0.0032; Fig. 3D).

**Figure 4:**
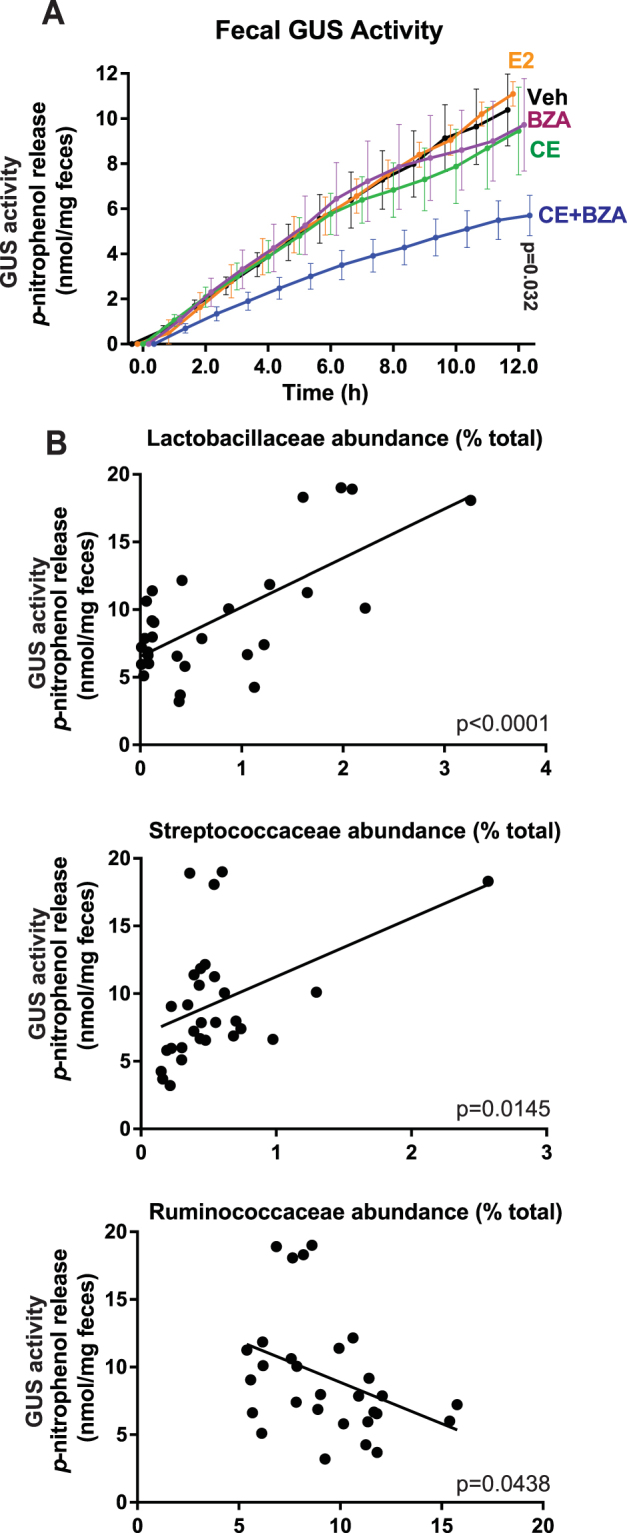
(**A**) Total GUS activity of fecal microbiota of mice (n = 8 per group) treated with: Veh - vehicle; E2 - estradiol; CE - conjugated estrogen; BZA - Bazedoxifene; CB - conjugated estrogen plus Bazedoxifene. *P < 0.05 compared to the activity of the control group at 12 h. Results are mean ± SEM. (**B**) Abundance of bacterial families in the fecal microbiome highly correlated (P < 0.05) with fecal GUS activity.

To further explore if long-term administration of estrogens impacted the GUS activity in the fecal samples, we performed GUS assays with the fecal lysate from animals that were treated with various estrogens using the colorimetric GUS substrate 4-nitrophenyl-β-D glucuronide (4-NPG). The total GUS activity, decreased significantly (P = 0.0293) in the group treated with conjugated estrogen combined with Bazedoxifene (Fig. 4A). Next, we focused on the correlation between GUS activity and the abundance of common bacterial families in the fecal microbiota. Further analysis identified a significant correlation between the abundance of Lactobacillaceae (P < 0.0001), Ruminococcaceae (P = 0.0335) and Streptococcaceae (P = 0.0138) and the fecal GUS activity (Fig. 4B).

### *In vitro* GUS activity in common gut bacteria

**Figure 5:**
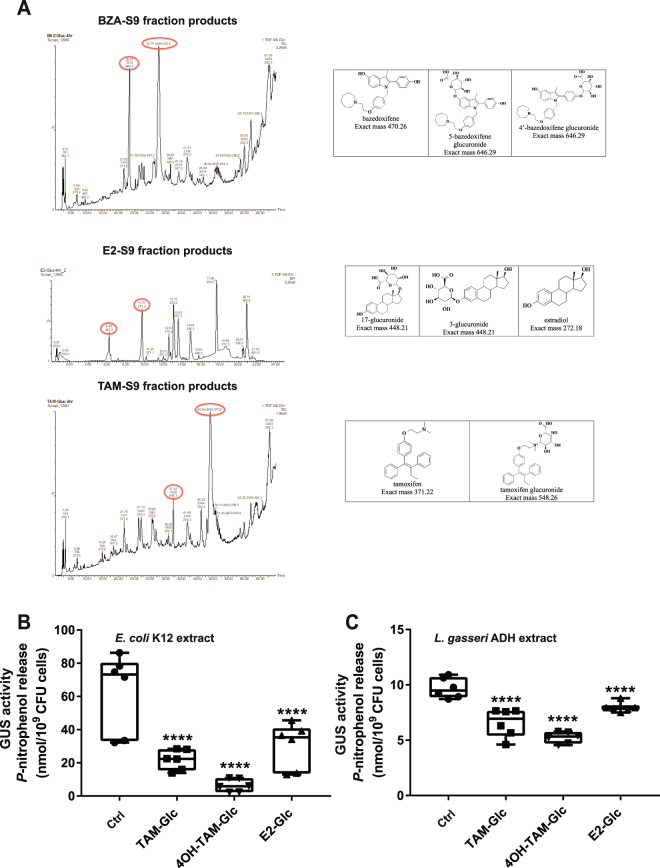
(**A**) S9 fraction glucuronidation metabolites of BZA, E2 and TAM. Competition between Tamoxifen-Glucuronide (TAM-Glc), 4-hydroxy-Tamoxifen-Glucuronide (4OH-TAM-Glc) or Estradiol-Glucuronide (E2-Glc) with GUS activity in (**B**) *Escherichia coli* K12 and (**C**) *Lactobacillus gasseri* ADH. Data showing min to max including three biological replicates and two technical duplicates in each treatment group (background corrected - ave. 1.9 nmol *p*-nitrophenol per 10^9^ CFU cells). Results are min to max. ****P < 0.0001 compared to the activity of the control group at 12 h.

To further explore how gut bacteria deconjugate estrogen glucuronides, we used an *in vitro* system which would be informative beyond the mouse. In this *in vitro* system, we have selected two well studied gut microorganisms as models, *Escherichia coli* K12 and *Lactobacillus gasseri* ADH. We examined *in vitro* whether these two model microorganisms, would directly deconjugate estrogen metabolites produced by human hepatic S9 fraction. First, the BZA, E2 and Tamoxifen (TAM) were incubated with the hepatic S9 fractions to ensure the formation of glucuronidated products. We identified 4- and 5-BZA-glucuronide, 17β- and 3-E2-glucuronide and Tam-glucuronide as products of glucuronidation reactions in liver (Fig. 5A). Next, to confirm whether the glucuronidated products were substrates for bacterial GUS enzymes, bacterial cell lysates were incubated with the colorimetric substrate, 4-NPG, with or without the addition of estrogen glucuronides, and the increase in A_405 nm_ from the release of the product *p*-nitrophenol was measured. Tamoxifen-, 4-hydroxy-Tamoxifen- and Estradiol-Glucuronide inhibited *p*-nitrophenol production with substrate 4-NPG at a ratio of 1:10, demonstrating a competition between this ligand and the hormonal substrates for the GUS enzymes in *E. coli* K12 and *L. gasseri* ADH (Fig. 5B and C).

## Discussion

In this study, for the first time we reported changes in gut microbial activity after long-term estrogen supplementation. We reported several bacteria taxa with GUS activity that changed upon CE+BZA administration. Exogenous estrogens are conjugated in the liver to increase their excretion. However, in the gut, bacteria with GUS enzymes hydrolyzes these conjugates, enabling these free estrogens that are released to enter systemic circulation and therefore increasing their circulatory half-life. While most studies report the variance in individual’s response to ERT^28^, the function of the gut microbiome, specifically the GUS activity of the gut microbiome, could have contributed to the effectiveness of ERT. To date, there has been compelling evidence that the gut microbiome affects level of systemic estrogens^21,29–31^. For example, enterohepatic circulation prolongs plasma estriol levels in postmenopausal women after receiving a single oral dose^31^. Women with decreased plasma concentration of estrogen were found to have reduced fecal GUS activity^32^. Of note, in this study, vegetarian and non-vegetarian women were compared. Their findings highlight the importance of diet composition in determining circulating estradiol levels in premenopausal women. The authors reported that vegetarian women had increased fecal output, increased fecal excretion of estrogens, and decreased level of circulating estrogen in the serum. Our mouse model of low estrogen and high-fat diet induced metabolic disease mimics the diet conditions for non-vegetarian women. Consistent with this finding, we observed that both serum glucuronic acid and fecal GUS activity decreased after CE+BZA treatment, which, for the first time, supports that the estrogen supplementation affects the gut microbiome composition and estrogen metabolism. Future clinical studies on postmenopausal women would be necessary to confirm this crosstalk between the diet, gut microbiome and ERT effectiveness.

The overall cecal or fecal microbiome community was not significantly altered by our analysis but the fecal GUS activity decreased following CE+BZA treatment. Although we identified that the abundance of *Akkermansia* decreased by CE+BZA, there could have been more gut microorganisms with altered abundance at the species- or strain-level, which was not discovered by the 16S rRNA sequencing. Yet importantly, we measured fecal GUS activity after estrogen supplementation to obtain a functional understanding of ERT on the gut microbiome. We also applied machine learning to integrate our previous metabolomic data into the microbiome analysis, which further advanced our knowledge on the crosstalk between the microbiome and the host. Additionally, the ERF approach identified *Akkermansia* associated with increased weight gain, liver weight and serum metabolites. Despite some studies reported an inverse correlation with Akkermansia and body weight^33^, our findings are consistent with several previously published studies on impact of hormone exposure and obesity^34,35^. One such study that reported inverse correlation was performed using only male mice^33^. There might be sex differences and hormonal influences that the authors did not observe in this study because of their experimental design. For example, in another study in utero BPA (an estrogenic compound) exposure caused weight gain and increased Akkermansia abundance in male mice (but not female mice)^34^. In addition, treatment with the hormones might change the abundance of the bacteria and might have direct causal effects on other biological functions (such as estrogen metabolism) rather than weight gain in female mice after ovariectomy and supplementation of estrogens. Finally, another study also reported increase in Akkermansia abundance with ovariectomy and weight gain in female mice^35^.

We found the correlation between fecal GUS activity and the relative abundance of the bacterial families Lactobacillaceae, Ruminococcaceae and Streptococcaceae in the fecal microbiota. Amongst these bacterial families, *Lactobacillus* has been shown to have GUS activity^36^. Although no species from Streptococcaceae have been reported to directly metabolize estrogens to date, a β-glucuronidase has been identified in *Streptococcus equi* subsp. Zooepidemicus^37^, suggesting that bacteria from the Streptococcaceae family may also contribute to the gut GUS activity. Future study on characterizing GUS enzymes from different bacterial families would be useful to advance our understanding on the role of gut microbiome in estrogen metabolism. For bacteria that lack GUS activity, their role in estrogen metabolism is unclear to date. However, analyzing the metabolites of different GUS-negative gut bacteria incubated with estrogens would elucidate if other enzymes would impact estrogen metabolism.

Although the *in vitro* GUS activity of *E. coli* K12 was higher than that of *L. gasseri* ADH, it should be noted that the abundance of *Escherichia* is <0.1% and was only detected in the ceca of two mice, while *Lactobacillus* was detected in nearly all the mice and constitutes 0.7% and 1.3% of the cecal and fecal microbiome, respectively. Also, the GUS activity of *Escherichia* and *Lactobacillus* in the gut may be different in the presence of other microorganisms and stresses such as bile salts^38,39^. Future *ex vivo* studies quantifying the transcripts of GUS genes in these microorganisms would provide insights on how ERT alters the expression of gut microbial GUS genes over time.

Overall, our observations implicate that gut microbial activity may affect an individual’s response to ERT. It may be possible that the microbiota metabolizes endogenous, synthetic or dietary estrogens to produce novel estrogen receptor ligands that can also be used to mitigate symptoms associated with menopause and obesity^40^. Alternatively, the gut microbiome may be manipulated to change half-life and properties of estrogens so that they could maintain metabolic function but reduce the risk of other diseases such as breast cancer. This is a significant because women who are diagnosed with breast cancer use Tamoxifen for 5 or 10 years^41^. A recent study showed that lower active TAM metabolite concentrations in the serum was associated with poor outcome^42^. In combination with our data, showing TAM and 4-OH-Tam as potential substrates for bacterial GUS activity, this study suggests combined probiotic interventions might improve the outcome of breast cancer patients using tamoxifen by increasing serum active TAM metabolites. The question remains as to whether bacterial composition in the gut changes in a way to affect half-life of these hormonal agents in the body. In addition, our study suggests that there may be an opportunity that through probiotic supplementation, we might be able to increase half-life of these hormonal agents in the body and effectively decrease the dose that the patient needs to take to achieve effective blood concentrations. Thus, the efficacy and safety of current ERT and other hormone therapy options could be optimized through the modulation of gut microbial activity by choosing the right combination and dosage of estrogens and/or dietary intervention by probiotic supplementation. Our study, for the first time provides the mechanistic basis for such approach to improve the health outcomes for postmenopausal women.

## Methods

### Animal model and treatments

All experiments involving animals were conducted with protocols approved by the University of Illinois at Urbana-Champaign and by the National Institutes of Health standards for use and care of animals (IACUC Protocol 14193). All the experiments were performed in accordance with relevant guidelines and regulations. Forty female C57BL/6 J mice were housed individually in 12-h light-dark cycle. All mice were given a high-fat diet and water *ad libitum* at 8 weeks of age and ovariectomized at 10 weeks of age then were divided randomly into five treatment groups as described previously^15^: (1) vehicle at 43% DMSO, 15% ethanol, and 42% saline (Veh); (2) E2 at 5 µg·kg^−1^·day^−1^; (3) CE at 2.5 mg·kg^−1^·day^−1^; (4) BZA at 3 mg·kg^−1^·day^−1^; (5) CE+BZA. The high fat diet for these studies (Harlan TD.88137) is based on an AIN76A diet (45% Kcal from fat). It is high in butterfat with sugar and some cholesterol (0.2%), and it was developed for metabolic studies in mice^43,44^. The very high fat diets (60% Kcal from fat) were not used to induce obesity, because we wanted to study a comparable level of fat consumed by humans in the US (~34% Kcal from fat; NHANES). We have previously used this diet to mimic the diet that humans in US consume and establish a model of low estrogen and high-fat diet associated metabolic disease in mice^13–15^. We selected the dosage of the estrogens based on previously published studies^9,10^. At the doses used, E2 and CE supplementation induced the predicted uterotropic responses whereas animals that were treated with Veh, BZA or CE+BZA did not display this response^15^. In addition, CE+BZA combination was very effective in preventing ovariectomy-induced weight gain and lipid deposition in liver^15^. Fecal and cecal samples were collected after 6 weeks of treatment.

### DNA extraction and 16S rRNA sequencing

Based on our previous work on body weight normalization by low affinity estrogens in mice treated with controls or various estrogens, typical treatment differences in the weight of animals on high fat diet following ovariectomy were 5 g; standard deviations were approximately 3 g^13^. Using these predictions and a Type I error of 5% and a Type II error of 10%, we estimated that 8 animals were required for each group in each experiment. At the end of six weeks of treatments with estrogens we were able to obtain high quality DNA from fecal and cecal samples of N = 8 Veh, N = 5 E2, N = 7 CE, N = 8 BZA and N = 6 CE+BZA treated mice. The 16S rRNA gene sequencing was performed by the DNA Sequencing Group at the Roy J. Carver Biotechnology Center, University of Illinois, using primers that amplified the V3-V5 variable regionsWater was used as the negative control to ensure that there was no non-specific amplification (0.006% non-specific amplification). Paired-end reads were generated on the Illumina MiSeq platform for each sample, at a read length of 250 nucleotides. The reads were demultiplexed and quality-filtered using Trimmomatic (version 0.30) with a Phred score cut-off of 33^45^. Paired-end reads were processed through IM-TORNADO (version 2.0.3.2)^46^. Operational taxonomical unit (OTU)-picking was performed through QIIME^47^ (version 1.9.0) by searching the SILVA ribosomal RNA database (128 release)^48^.

### Calculation of diversity measures and correlation analysis

The bacterial genera in the cecal and fecal microbiome was compared in a Venn diagram (Venny 2.1.0; http://bioinfogp.cnb.csic.es/tools/venny/). The alpha-diversity (Shannon and the Simpson index) was calculated from a rarefied OTU table^49^. Clustering analysis was performed based on weighted Unifrac distances. Pearson correlation was performed on the abundance of fecal bacterial families with GUS activity.

### Extended Random Forest

Extended Random Forest was applied to identify and rank OTUs predictive of the percent weight gain. By “extending” the original data set with random permutated features and applying the Random Forest algorithm stability of all relevant OTUs is ensured^25^. The microbiome-metabolome metadata was pre-processed by dropping features with zero variance, and we incorporated a parameter-space mapping algorithm to determine the model parameters for RF. Using these parameters (Ntree: 10000, Mtry: 12), RF constructed an ensemble of classification from bootstrap samples of the data. Each tree was fit using a random subset of features to define the best split at each node that allowed for trees to be fit in a lower dimension without introducing additional bias while the averaging of these trees removed variance from the prediction. The “out-of-bag” (OOB) samples, i.e., observations left out of the bootstrap samples, were used to estimate prediction error of trees. The variable importance measure (VIM) for feature X_j_, was the difference in prediction error caused by randomly permuting the X_j_ OOB sample values. Therefore, VIM represents the benefit of having a feature included in a tree compared to random noise. For feature ranking, the RF permutation-accuracy measure was used to capture the impact of each feature individually, as well as conditional importance from multivariate interactions between the features^50^.

### Bacterial strains and culture conditions

*Escherichia coli* K12 (American Type Culture Collection, VA), was grown in Luria-Bertani (LB) broth (BD Difco, MD) at 37 °C with shaking at 200 rpm. *Lactobacillus gasseri* ADH (generous gift from the Klaenhammer Lab, North Carolina State University)^51^, was grown in De Man, Rogosa, Sharpe (MRS) broth (Thermo Fisher Scientific, NJ) at 37 °C.

### S9 Fraction metabolism

To validate formation of glucuronidated estrogen metabolites by Phase 2 metabolism, human S9 fraction (S2442; Sigma-Aldrich, MO) with a glucuronidation cofactor, uridine 5′-diphosphoglucuronic acid (UDPGA) (U6751; Sigma-Aldrich, MO) was used^52^. S9 fraction (20 mg/mL) was incubated with 3 mM of BZA, E2 or TAM and 4 mM UDPGA at 37 °C for 4 h. The reaction was stopped by adding 0.6 ml MeOH to 0.2 mL of reaction mix. Samples were centrifuged at 10,000 rpm for one minute and dried. Parent compounds and glucuronidated products were detected using LC-MS.

### GUS activity and competition by estrogen glucuronides in model microorganisms

Chemicals were purchased from Santa Cruz Biotechnology (TX) unless specified. The GUS activity in fecal microbiota and model microorganisms was monitored by the absorbance at 405 nm from the *p-*nitrophenol release using 4-nitrophenyl β-D-glucopyranoside (4-NPG) as the substrate.

The frozen fecal pellets were homogenized in PBS, and the total protein concentration was determined using Pierce™ BCA Protein Assay (Thermo Fisher Scientific, NJ). Fecal homogenate from all treatment groups containing 200 µg/mL protein was incubated (1:1 v/v) with 1 mM 4-NPG at 30 °C for 12 h. The A_405 nm_ was monitored and the *p-*nitrophenol release was calibrated against a standard curve. The GUS activity from fecal samples was expressed as the amount of *p-*nitrophenol released per mg of faeces.

The 24-h cultures of *E. coli* K12 and *L. gasseri* ADH were pelleted at a table-top centrifuge (10,000 xg for 30 sec) and resuspended in phosphate buffered saline (PBS) to optical density (OD_600 nm_) = 1. Cells were homogenized using a horizontal vortex adaptor for 3 min, and the debris was removed by pelleting in a table-top centrifuge (10,000 × g for 30 sec). The supernatant was kept at −20 °C or on ice prior to the GUS assays. The GUS activity from the bacterial culture was expressed as the amount of *p-*nitrophenol released per 10^9^ colony forming unit (CFU).

For competition assays, stock solutions of (E,Z)-Tamoxifen N-β-D-Glucuronide (TAM-Glc), (Z)-4-Hydroxy tamoxifen O-β-D-glucuronide (4OH-TAM-Glc) and β-Estradiol 17β-D-glucuronide (E2-Glc) sodium salt were prepared in dimethyl sulfoxide (Cambridge Isotope Laboratories, MA), and diluted in PBS to final 125 µM and incubated with 1.25 mM 4-NPG at 30 °C for 12 h. The experiment was repeated including three biological replicates and two technical duplicates.

### Statistical Analyses

The percentage total of cecal and fecal bacterial taxa at different phylogenetic levels following different treatments were analysed using one-way analysis of variance (ANOVA) model. Data from GUS activity were analysed using a two-way ANOVA model to time-dependent treatment effects. For every main effect that was statistically significant at 0.05, pairwise t-tests were conducted to determine which ligand or inhibitor treatment levels were significantly different from each other. For these t-tests, the Bonferroni correction was employed to control the experimentwise type I error rate at α = 0.05 followed by Bonferroni post hoc test (GraphPad Prism version 7.03; GraphPad Software, La Jolla California USA, www.graphpad.com).

## Electronic supplementary material

Supplementary table 1
