# Supplementary material for: Long-Term Administration of Conjugated Estrogen and Bazedoxifene Decreased Murine Fecal β-Glucuronidase Activity Without Impacting Overall Microbiome Community
Source: Sci Rep. 2018 May 25;8:8166. doi: 10.1038/s41598-018-26506-1 (PMC5970144; doi:10.1038/s41598-018-26506-1)
Supplement: Supplementary file 1 — Supplementary table 1 [file 41598_2018_26506_MOESM1_ESM.doc]

# Supplementary Table

# Long-Term Administration of Conjugated Estrogen and Bazedoxifene Decreased Murine Fecal β-Glucuronidase Activity Without Impacting Overall Microbiome Community

Karen Lee Ann Chen^1*^, Xiaoji Liu^2*^, Yiru Chen Zhao^2^, Kadriye Hieronymi^2^, Gianluigi Rossi^3,+^, Loretta Sue Auvil^4^, Michael Welge^4,5^, Colleen Bushell^4,5,6^, Rebecca Lee Smith^3,4,5^, Kathryn E. Carlson^7^, Sung Hoon Kim^7^, John A. Katzenellenbogen^7^, Michael Joseph Miller^1,2^, Zeynep Madak-Erdogan^1,2,4,5,8,9^

# Supplementary Table S1. List of bacterial genera highly correlated between the cecal and fecal microbiome of ovariectomized mice treated with: Veh - vehicle (n=8); E2 - estradiol (n=5); CE - conjugated estrogen (n=7); BZA - Bazedoxifene (n=8); CE+BZA - conjugated estrogen combined with Bazedoxifene (n=6).

| Genus | P-value |
| --- | --- |
| *Enterobacteriaceae;g__* | 0.000 |
| *Enterobacteriaceae;Other* | 0.000 |
| *Parabacteroides* | 0.000 |
| *Sutterella* | 0.000 |
| *Lachnospiraceae;g__* | 0.000 |
| *Bacteroides* | 0.000 |
| *Coprococcus* | 0.000 |
| *Ruminococcaceae;g__* | 0.000 |
| *Mogibacteriaceae;g__* | 0.000 |
| *Erysipelotrichaceae;g__* | 0.000 |
| *Clostridiaceae;g__* | 0.000 |
| *Coriobacteriaceae;g__* | 0.000 |
| *Staphylococcus* | 0.000 |
| *Ruminococcus* | 0.000 |
| *Anaerostipes* | 0.001 |
| *Akkermansia* | 0.002 |
| *02d06* | 0.002 |
| *Christensenellaceae;g__* | 0.002 |
| *Dehalobacterium* | 0.004 |
| *Bacillales;Other* | 0.006 |
| *Blautia* | 0.008 |
| *Firmicutes;Other* | 0.008 |
| *Peptostreptococcaceae;Other* | 0.013 |
| *Clostridiaceae;Other* | 0.018 |
| *Bacteria;Other* | 0.018 |
| *Peptostreptococcaceae;g__* | 0.020 |
| *Turicibacter* | 0.031 |
